# The Difference of Chemical Components and Biological Activities of the Crude Products and the Salt-Processed Product from Semen Cuscutae

**DOI:** 10.1155/2016/8656740

**Published:** 2016-08-16

**Authors:** Song Yang, Hefang Xu, Baosheng Zhao, Shasha Li, Tingting Li, Xinfang Xu, Tianjiao Zhang, Ruichao Lin, Jian Li, Xiangri Li

**Affiliations:** ^1^School of Chinese Materia Medica, Beijing University of Chinese Medicine, No. 6 Wangjing Zhonghuan Nan Road, Beijing 100102, China; ^2^School of Basic Medical Sciences, Beijing University of Chinese Medicine, No. 11 North Third Ring Road, Beijing 100029, China; ^3^Beijing Key Laboratory of Chinese Materia Medica Quality Evaluation, Beijing University of Chinese Medicine, No. 11 North Third Ring Road, Beijing 100029, China

## Abstract

Semen Cuscutae is a well-known Chinese medicine which has been used to nourish kidney in China for thousands of years. The crude product of semen Cuscutae (CP) and its salt-processed product (SPP) are separately used in clinic for their different effects. The study was designed to investigate the influence of processing from semen Cuscutae on chemical components and biological effects. The principal component analysis and quantitative analysis were used to study the differences of the chemical components. The effects of nourishing kidney were detected to compare the differences between the CP and SPP. The PCA results showed that the obvious separation was achieved in the CP and SPP samples. The results of quantitative analysis showed that quercetin and total flavonoids had significantly increased after salt processing while hyperoside had decreased. The comparison of CP and SPP on biological activities showed that both of them could ameliorate the kidney-yang deficiency syndrome by restoring the level of sex hormone, improving the immune function and antioxidant effect. However, SPP was better in increasing the level of T and the viscera weight of testicle and epididymis, improving the antioxidant effect. The results suggested that salt processing changed its chemical profile, which in turn enhanced its biological activities.

## 1. Introduction

Semen cuscutae, the dry matured seed of* Cuscuta chinensis *Lam. or* Cuscuta australis* R. Br., is an important Chinese medicine first recorded in the “Shen Nong's Herbal” as an upper grade drug. It has been used to nourish the liver and kidney, treat impotence and seminal emission, prevent miscarriage, and improve eyesight in China for thousands of years. The main constituents of semen cuscutae have been shown as various flavonoids compounds, a range of polysaccharides, a number of different alkaloids, and various other chemicals [1–4]. The flavonoids and polysaccharides are the main biologically active constituents in semen cuscutae. Modern pharmacological experiments have indicated that semen cuscutae could reverse the reduction in testosterone level and the expression of androgen receptor gene, possess immunomodulatory activities, hepatoprotective effects, antioxidation effect, anti-inflammatory action, and antiaging effects, and enhance memory by inducing PC12 cell differentiation [5–8].

**Figure 1:**
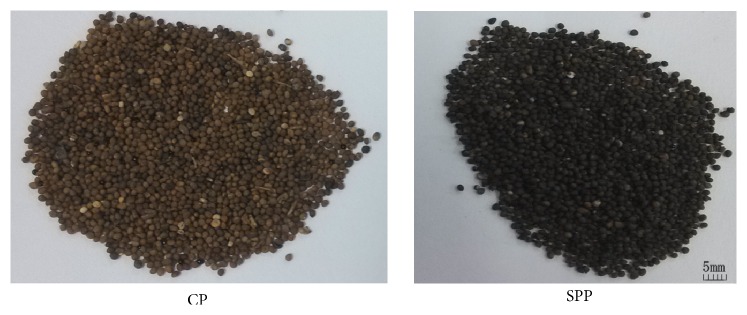
The crude product of semen cuscutae (CP) and the salt-processed product (SPP).

In traditional Chinese medicine, the processing of traditional Chinese herbs is a common practice and usually occurs before prescription [9]. Medicinal plants sometimes require specific processing steps such as cleaning, water processing (grinding in water, steaming, and roasting), and fire processing (stir-heating with wine, vinegar, salt, or honey) [10, 11]. The main purposes of processing are to reduce toxicity and to enhance the effects of crude drugs. The chemical components of crude drugs and processed products are different: new components may be formed or the relative contents of certain components may change; other components may disappear or their contents may decrease [12]. According to the theory of traditional Chinese medicine (TCM), herbs processed with salt manifest their effects on kidney meridian [13]. Processing semen cuscutae has a long history, and salt-processed product (SPP) which is processed by stir-heating with salt solution is documented in the 2015 edition of Chinese pharmacopoeia. SPP is first documented in “Comprehensive Recording of Sage-Like Benefit” (Sheng Ji Zong Lu) in the North Song Dynasty of China and then the crude products of semen cuscutae (CP) and SPP are separately used clinically for their different effects. The CP and SPP were shown in Figure 1. SPP is better in warming and tonifying kidney-yang which was documented in “Combinations of Materia Medica” in the Qing Dynasty of China [14]. However, up to now, the differences of chemical components between CP and SPP have not been studied.

Modern pharmacological researches have indicated that the semen cuscutae could tonify the kidney to secure essence and is used to treat the diseases of deficiency in the kidney such as deficiency of kidney-yang [15, 16]. However, there were no comparative studies on the biological activities between CP and SPP so far. Recently, to facilitate the experimental research, the kidney-yang deficiency animal model has been duplicated by injecting rats with a high dose of hydrocortisone, in which rats will show symptoms greatly resembling those described in TCM kidney-yang deficiency [17]. Rats with kidney-yang deficiency always have some symptoms such as raritas clothing hair, weight loss, slowed reaction, aversion cold, weakness, tendency to cluster, and decreased activity. Based on the common kidney-yang deficiency animal model, the effects of nourishing kidney were detected to compare the differences between CP and SPP and further to unveil the scientific essence of TCM processing.

## 2. Materials and Methods

### 2.1. Materials and Chemicals

Hyperoside, quercetin, astragalin, kaempferol, and isorhamnetin were purchased from National Institutes for Food and Drug Control. The purities of all the standards were no less than 98%. Methanol (HPLC grade) was purchased from Thermo Fisher Scientific Inc. Deionized water was prepared by a MilliQ50 SP Reagent Water System (Bedford, MA, USA) for preparing samples and mobile solution. Other reagents were of analytical grade. SOD and MDA kits were purchased from Nanjing Jiancheng Bioengineering Institute. CD3-FITC fluorescent antibody, CD4-APC fluorescent antibody, and CD8a-PE fluorescent antibody were purchased from BD Company (USA).

### 2.2. Samples Collection

All the crude products of semen cuscutae (CP, 6 samples) were collected by Beijing University of Chinese Medicine. The samples of the crude products were collected from Heilongjiang and Neimeng provinces, respectively, which were identified as* Cuscuta australis* R. Br. by Yang Yaojun, the professor of Pharmacognosy Department in Beijing University of Chinese Medicine. Voucher specimens were deposited at the School of Chinese Materia, Beijing University of Chinese Medicine.

SPP samples were prepared in the lab, which means that the CP was mixed with salt solution and then stir-heated at 80–100°C in a metallic pan and dried in the air.

### 2.3. Principal Component Analysis (PCA)

To compare the differences between CP and SPP, unsupervised principal component analysis was performed based on the relative peak areas in the HPLC fingerprints chromatography using software of Umetrics EZinfo. The main chemical markers which have the most influence on the classification among different samples were found out with the help of PCA loading Bi plots.

### 2.4. Sample Preparation

#### 2.4.1. Extract Preparation for Animals

The CP and SPP were selected from the samples we collected and prepared in our lab in Section 2.2.

The CP was accurately weighed as 1.0 kg and soaked 8 times in 60% ethanol for one night. And then, it was extracted under reflux twice for 1 h each time. The filtrate was combined and ethanol was recovered. The total crude flavonoids extract was dried in vacuum at 60°C. The crude extract was purified by macroporous resin. Then, the flavonoids extract of CP was obtained.

The flavonoids extract of SPP was obtained following the same procedure above.

The flavonoids extracts of CP and SPP were mixed suspension in water, respectively, when the rats were treated orally.

#### 2.4.2. Sample Preparation for HPLC

The flavonoids extracts in Section 2.4.1 were accurately weighed as 0.15 g and extracted with 50 mL 80% methanol under reflux for 2 h. After cooling, the loss of weight was replenished with 80% methanol. All solvents and samples were filtered through 0.45 *μ*m membrane before analysis. The contents of 2 flavonoids in CP and SPP were determined using HPLC.

#### 2.4.3. Sample Preparation for Determining the Total Flavonoids

The flavonoids extracts in Section 2.4.1 were accurately weighed as 0.05 g and extracted with 40 mL ethanol under reflux twice for 1 h each time. The filtrate was combined in 100 mL volumetric flask.

### 2.5. Chromatographic Condition of HPLC Fingerprints and Determining the 2 Flavonoids

The chromatographic separation was performed in a Waters LC system (Waters Technologies, USA), equipped with a 1525 binary pump, a column compartment, and a 2489 double wavelength UV detector. The samples were separated on an Agilent Zorbax SB-C18 (250 mm × 4.6 mm, 5 *μ*m) with a mobile phase that consisted of methanol (A) and 0.4% phosphoric acid solution (B) at a flow rate of 1 mL/min. The gradient elution employed was 25%–40% A at 0–60 minutes and then 40%–60% A at 60–80 minutes. The temperature was maintained at an obligatory level of 40°C. The wavelength of the detector was kept at 365 nm. The sample volume injected was 15 *μ*L. The major constituents in the multiple extracts and fractions were identified by comparing their retention time with the flavonoid standards.

The chromatographic condition which was described above had been established by our team [18].

### 2.6. Determination of Contents of Total Flavonoids

The condition had also been established by our team [19]. The total flavonoids contents of the CP and SPP were determined by the NaNO_2_-Al(NO_3_)_3_-NaOH method.

### 2.7. Animal Experiments

#### 2.7.1. Animals and Housing

SD male rats (purchased from Vital River Laboratories Co., Ltd.) weighing 200 ± 20 g (age: 6 weeks old) were separated into different groups by randomized procedure and acclimatized for 1 week prior to treatment. The rats were maintained under standard laboratory conditions (temperature of 21–23°C, relative humidity of 45–65%, and 12 h/12 h light/dark cycle) with food and water freely available. All animal experiments were performed according to the ethical guidelines suggested by the Institutional Animal Ethics Committee and Committee for the Purpose of Control and Supervision of Experiments on Animals, Ministry of Health, and Government of China.

#### 2.7.2. Treatment of Animals

The rats were partitioned into 4 groups (*n* = 10 in each group); they were given an intramuscular injection of 20 mg/kg hydrocortisone sodium succinate (purchased from Tianjin Biochem Pharmaceutical Co., Ltd., Tianjin, China) for 14 days except for group 1 (normal group, N) which were injected with an equal volume of physiological saline. After this treatment, the kidney-yang deficiency model was made. On the 15th day, 4 groups were treated as follows: group 3 (CP group, CP) were treated orally at dose of 0.14 g/kg flavonoids extract of CP, group 4 (SPP group, SPP) were treated orally with 0.15 g/kg flavonoids extract of SPP, and group 1 and group 2 (model group, M) were treated orally with equal volume distilled water. All rats were treated by gastric perfusion. After 4 weeks' treatment, the rats were denied food for 12 h before blood collection. On the next day, all rats were anesthetized and sacrificed. Blood samples were first collected from eye angular vein to EP tubes of EDTA anticoagulant and then CD4^+^ (%), CD8^+^ (%), and CD4^+^/CD8^+^ were determined by FACS2Calibur FCM (BD Company, USA). The blood samples from abdominal aorta had been centrifuged (1500 rpm, 15 min) to obtain the serum. The serum samples were stored at −80°C until the level of sex hormones, IgG and IgM, was determined. One kidney and one testicle were rapidly removed and weighed as well as epididymis, prostate gland, and seminal vesicle. The other testicle was frozen in liquid nitrogen for the determination of SOD and MDA.

#### 2.7.3. Weight Analysis

During the testing, the rats were weighed once a week for adjusting the dosage and weighed before sacrificing.

### 2.8. Hormone, Immunization, and Antioxidant Effect Analysis

Before the rats were executed, their serum was collected. Total serum testosterone (T), estradiol (E_2_), IgG, and IgM level was determined in the Beijing North Biotechnology Research Institute by radioimmunity and colorimetric method. CD4^+^ (%), CD8^+^ (%), and CD4^+^/CD8^+^ were determined by FACS2Calibur FCM (BD Company, USA) in Scientific Research Center of BUCM.

The frozen testicles were thawed and weighed. Then, they were mixed with cold physiological saline. The ratio of testicle and physiological saline is 1 : 9 (W/W). The testicle homogenate which was obtained by tissue homogenate in the ice bath was centrifugated to obtain the supernatant. The contents of SOD and MDA of testicle were determined by SOD and MDA kits, following the instructions.

### 2.9. Statistical Analysis

All data was expressed as mean ± standard deviations and analyzed with one-way analysis of variance (ANOVA). Scheffe's test was used to calculate statistical significance by SPSS software 17.0. *P* < 0.05 was considered statistically significant.

## 3. Results 

### 3.1. The Analysis of Chemical Components Change during Processing with Salt Solution from PCA

**Figure 2:**
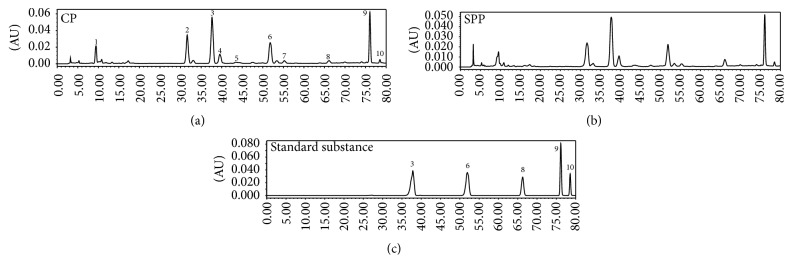
HPLC chromatogram (3: hyperoside, 6: astragalin, 8: quercetin, 9: kaempferol, and 10: isorhamnetin).

**Figure 3:**
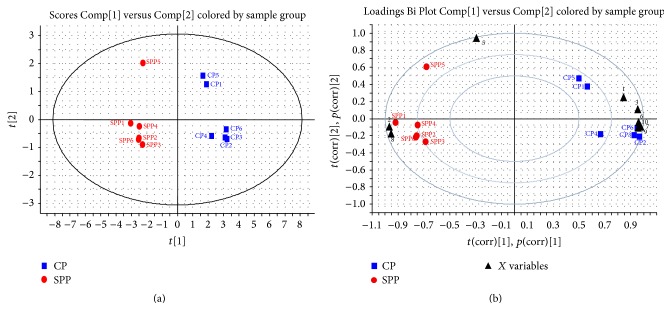
PCA score plot (a) and loading Bi plot (b) of CP (blue box) and SPP (red dot). CP and SPP were classified into two clusters. Peaks 3, 6, 7, 9, 10, and 1 are the most important components to distinguish CP and SPP, which will reduce during processing; peaks 2 and 8 are also the most important components for the differences between CP and SPP, which will increase during processing.

According to our early work, there were no new components after processing with salt solution but the contents change occurred during the processing, and 10 components were identified [20]. In this paper, 10 chromatographic peaks were selected as characteristic peaks, the relative peak areas (RPA) of which were calculated for quantitative expression of the HPLC fingerprints. The HPLC chromatogram was shown in Figure 2. PCA analysis on the RPA of 10 components was obtained for discrimination of different samples. For the UV data set by the first two principal components, the plot of the scores (Figure 3(a)) indicated that the samples were classified into two clusters. In the scores plot obtained by PCA, CP and SPP were farther from each other. It is believed that the contents of the chemical constituents in CP and SPP are different. To find the potential chemical markers for the discrimination between CP and SPP, the extended statistical analysis was performed to generate the loading Bi plot (Figure 3(b)). Peaks 3, 6, 7, 9, 10, and 1 are the most important components to distinguish CP and SPP, which will reduce during processing. Moreover, peaks 2 and 8 are also the most important components for the differences between CP and SPP, which will increase during processing. The relative contents of peak 5 will not vary much during processing. As shown in Figure 2, peak 8 was quercetin and peak 3 was hyperoside.

### 3.2. The Contents of Quercetin and Total Flavonoids Had Increased after Salt Processing

Next, the corresponding “loading Bi plot” analysis was used to visualize which components have changed a lot. As shown in Figure 3(b), the result showed that 8 components (2 and 8 increased and 3, 6, 7, 9, 10, and 1 decreased) had a major impact on the sample clustering which had changed significantly. Among them, quercetin and quercetin glucosides (quercetin and quercetin-3-O-*β*-D-gal(2-1)*β*-D-api) had increased the most while hyperoside decreased. So, the contents of quercetin and hyperoside were determined by HPLC.

The total flavonoids in CP and SPP were determined by NaNO_2_-Al(NO_3_)_3_-NaOH method. As shown in Table 1, quercetin and total flavonoids in SPP had increased significantly compared with CP. Hyperoside in SPP had decreased significantly.

### 3.3. The Kidney-Yang Deficiency Model Was Successfully Made

After 14 days' intramuscular injection, the rats showed raritas clothing hair, weight loss, slowed reaction, aversion cold, weakness, tendency to cluster, and decreased activity as in kidney-yang deficiency. Compared with normal group, the T and E_2_ level in the serum of the model group was lower. From what has been discussed above, the kidney-yang deficiency model was successfully made.

**Figure 4:**
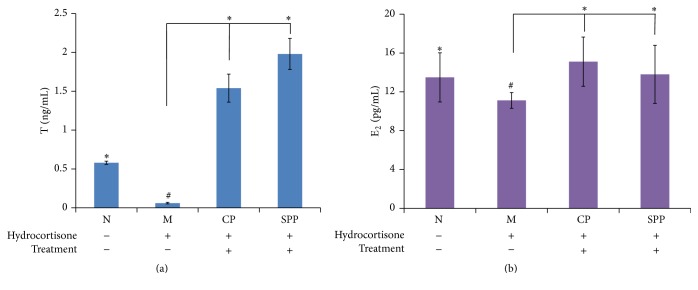
(a) and (b) were the level of T and E_2_. Compared with M group, the levels of T and E_2_ in CP and SPP groups both had increased; however, SPP group was better than CP group. Significant differences with M group were designated as ^*∗*^ *P* < 0.05. Significant differences with N group were designated as ^#^ *P* < 0.05.

On the other hand, the level of T and E_2_ in other treated groups had increased as shown in Figure 4, and the SPP group was better than CP group in the level of T.

### 3.4. The Average Viscera Weight and Index of CP and SPP Group

During making the model of kidney-yang deficiency, some of the rats were dead. We weighed all rats and their viscera and then calculated the average viscera index.

Compared with the normal group, the viscera weights of kidney, epididymis, seminal vesicle, and prostate gland were decreased in model group. The SPP group had increased weight of kidney, testicle, epididymis, and seminal vesicle. The CP group had increased weight of kidney, epididymis, seminal vesicle, and prostate gland. The SPP group was better in testicle and epididymis than CP group. The results were shown in Table 2.

Compared with the normal group, the viscera indexes of testicle, epididymis, seminal vesicle, and prostate gland were decreased in model group. The SPP group had the most increase in testicle and epididymis indexes. The CP group had the greatest increase in seminal vesicle index. The SPP group was better in testicle and epididymis indexes than CP group. The results were shown in Table 3.

### 3.5. The Influences of CP and SPP on Immune System in Kidney-Yang Deficiency Rats

**Figure 5:**
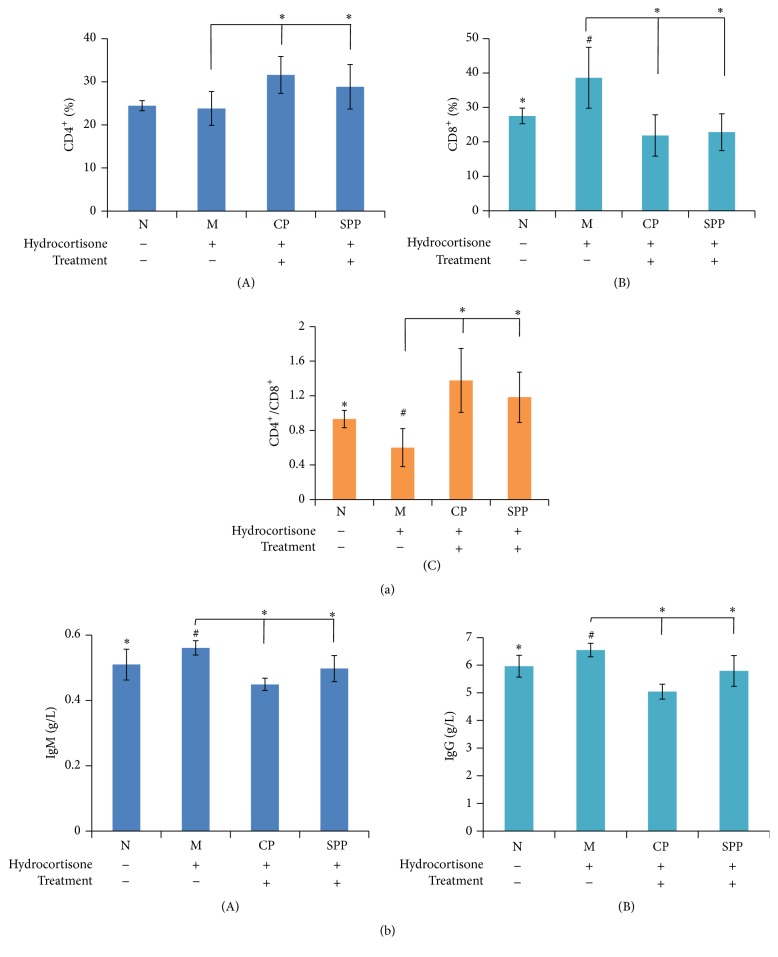
The results of immune activity in each group of rats. (a) T cells subtype detection by FCM ((A) CD4^+^, (B) CD8^+^, and (C) CD4^+^/CD8^+^). (b) The contents of IgM and IgG in serum. Compared with M group, the percentage of CD8^+^ had decreased while CD4^+^/CD8^+^ increased in CP and SPP groups. The contents of IgM and IgG also decreased. Significant differences with M group were designated as ^*∗*^ *P* < 0.05. Significant differences with N group were designated as ^#^ *P* < 0.05.

T lymphocytes are active cells of immune system in the body; their activation, differentiation, and proliferation play an important role in the process of immune response. Balanced state of Th1/Th2 is the precondition of immune stable internal environment. As shown in Figure 5(a), compared with normal group, the T lymphocyte subsets were in the condition of inhibiting the expression and CD8^+^ (%) was increased while CD4^+^/CD8^+^ decreased significantly in model group. All treated groups had improved this condition. CP and SPP can regulate T lymphocyte subgroup level and improve the immune function.

Immunoglobulin is an important immune molecule of the body, and the contents of IgG and IgM respond to the immune ability to some extent. As shown in Figure 5(b), compared with the normal group, the contents of IgG and IgM were increased in model group; the CP and SPP groups had decreased level of IgG and IgM.

### 3.6. SPP and CP Groups Both Showed Antioxidant Effect

**Figure 6:**
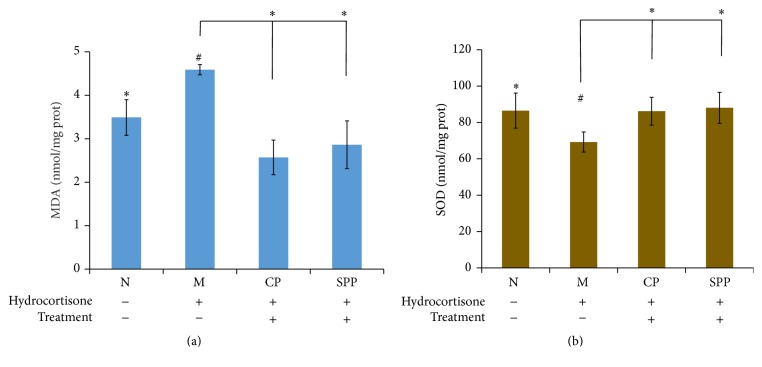
(a) and (b) were the contents of MDA and SOD. Compared with M group, the antioxidant effect in CP and SPP groups had been enhanced. Significant differences with M group were designated as ^*∗*^ *P* < 0.05. Significant differences with N group were designated as ^#^ *P* < 0.05.

Malondialdehyde (MDA) is the product of lipid peroxidation which is the indicator of reflecting the degree of oxidant damage. Superoxide dismutase (SOD) is an important antioxidant enzyme. The contents of MDA and SOD respond to the extent of oxidant and antioxidant ability. As shown in Figure 6, SPP and CP both had antioxidant effect.

## 4. Discussion

Prior to their usage in clinics, crude drugs are subjected to traditional Chinese processing techniques. SPP as one of the processing products of CP is better in terms of nourishing kidney which was documented in the 2015 edition of Chinese pharmacopoeia. In our study, the PCA results indicated that the chemical profile of CP changed after salt processing. What is more, 8 components had a major impact on the sample clustering which had changed significantly. Among them, quercetin and total flavonoids in SPP had increased most while hyperoside decreased. The findings in this study demonstrated for the first time that salt processing could change chemical constituents of crude products. Hydrolysis reaction might happen in processing, which led to the change of chemical constituents.

The flavonoids in semen cuscutae could reverse the reduction in testosterone level and possess immunomodulatory activities, antioxidant effect, and anti-inflammatory action [5, 21, 22]. The quercetin, as one of the flavonoids, has antioxidation and anti-inflammatory effects [23, 24]. So, the comparison of pharmacological effects in testosterone level, immunomodulatory activities, antioxidation effect, and anti-inflammatory effect of CP and SPP was studied. In the model of kidney-yang deficiency, the endocrine was disrupted by hydrocortisone, and the level of sex hormone was changed in the blood. Hydrocortisone is also a kind of immunosuppressant and long-term injection can cause the body's immune function disorder. In our study, the sex hormone analysis results showed that both CP and SPP groups had increased level of T and E_2_ effectively, and SPP is better than CP. The results of the viscera indexes showed that SPP is better than CP in increasing the weights and indexes of testicle and epididymis. In the aspect of the immune system, CP and SPP both could improve the immune function through regulating T lymphocyte subgroup level and decreasing the level of IgG and IgM. In addition, semen cuscutae has a significant antioxidant effect, especially SPP. From what we discussed above, CP and SPP both could improve the kidney-yang deficiency syndrome; however, SPP is better than CP in increasing the level of T and the weights and indexes of testicle and epididymis. The results of the pharmacological effects study indicated for the first time that salt processing changed the amount of flavonoids, which in turn increased the sex hormone level, improved immune function, and enhanced antioxidant effect.

## 5. Conclusion

The study was to discover the change of the flavonoids and the pharmacological effect in CP and SPP on the kidney-yang deficiency rat, especially on hormone level and antioxidant effect. It is important to note that this study has demonstrated that processing of traditional Chinese herbs is able to alter their chemical constituents and affect their bioactivity profile. It is also supported that crude products and processed products were prescribed differently in clinic.

## Figures and Tables

**Table 1:** The contents of total flavonoids, hyperoside, and quercetin in CP and SPP (mg/g) ($\bar{x}\pm s$).

	Total flavonoids	Hyperoside	Quercetin
CP	19.84 ± 0.009	2.84 ± 0.021	0.09 ± 0.001
SPP	20.22 ± 0.005^*∗*^	2.65 ± 0.007^*∗*^	0.16 ± 0.001^*∗*^

Note: significant differences with CP were designated as ^*∗*^ *P* < 0.05.

**Table 2:** The viscera weight (g) ($\bar{x}\pm s$).

	N	M	CP	SPP
Kidney	1.1941 ± 0.0500^*∗*^	1.0494 ± 0.0377^#^	1.3440 ± 0.0516^*∗*^	1.3030 ± 0.0824^*∗*^
Testicle	1.5360 ± 0.0655	1.4441 ± 0.0396	1.1320 ± 0.0346	1.8201 ± 0.0260^*∗*^
Epididymis	0.4325 ± 0.0307^*∗*^	0.3533 ± 0.0177^#^	0.5409 ± 0.3200^*∗*^	0.5534 ± 0.0174^*∗*^
Seminal vesicle	0.7724 ± 0.0420^*∗*^	0.6434 ± 0.0719^#^	1.9516 ± 0.1142^*∗*^	1.5731 ± 0.0777^*∗*^
Prostate gland	0.5919 ± 0.0258^*∗*^	0.4022 ± 0.0599^#^	0.6241 ± 0.0787^*∗*^	0.4934 ± 0.0443

Note: significant differences with M group were designated as ^*∗*^ *P* < 0.05. Significant differences with N group were designated as ^#^ *P* < 0.05.

**Table 3:** The average viscera index ($\bar{x}\pm s$).

	N	M	CP	SPP
Kidney	0.3852 ± 0.0303	0.3656 ± 0.0310	0.3277 ± 0.0214^*∗*^	0.3777 ± 0.0424
Testicle	0.4978 ± 0.0396^*∗*^	0.4139 ± 0.0211^#^	0.4594 ± 0.0246^*∗*^	0.4981 ± 0.0363^*∗*^
Epididymis	0.1413 ± 0.0155^*∗*^	0.1261 ± 0.0119^#^	0.1317 ± 0.0213	0.1557 ± 0.0208^*∗*^
Seminal vesicle	0.4580 ± 0.0268^*∗*^	0.4098 ± 0.0114^#^	0.4735 ± 0.0597^*∗*^	0.4516 ± 0.0416^*∗*^
Prostate gland	0.1923 ± 0.0304^*∗*^	0.1231 ± 0.0361^#^	0.1514 ± 0.0119^*∗*^	0.1330 ± 0.0341

Note: significant differences with M group were designated as ^*∗*^ *P* < 0.05. Significant differences with N group were designated as ^#^ *P* < 0.05.

## References

[1] Ye M., Yan Y.-N., Qiao L., Ni X.-M. (2002). Studies on chemical constituents of Cuscuta chinensis. *China Journal of Chinese Materia Medica*.

[2] Du X.-M., Kohinata K., Kawasaki T., Guo Y.-T., Miyahara K. (1998). Components of the ether-insoluble resin glycoside-like fraction from *Cuscuta chinensis*. *Phytochemistry*.

[3] Miyahara K., Du X.-M., Watanabe M., Sugimura C., Yahara S., Nohara T. (1996). Resin glycosides. XXIII. Two novel acylated trisaccharides related to resin glycoside from the seeds of Cuscuta chinensis. *Chemical and Pharmaceutical Bulletin*.

[4] Yahara S., Domoto H., Sugimura C. (1994). An alkaloid and two lignans from *Cuscuta chinensis*. *Phytochemistry*.

[5] Lin M. K., Lee M. S., Chang W. T., Yang M. C., Chu C. L. (2009). Study the immunomodulatory activity of semen cuscutae and identify the active components. *Journal of Bioscience and Bioengineering*.

[6] Liao J.-C., Chang W.-T., Lee M.-S. (2014). Antinociceptive and anti-inflammatory activities of cuscuta chinensis seeds in mice. *The American Journal of Chinese Medicine*.

[7] Yen F.-L., Wu T.-H., Lin L.-T., Lin C.-C. (2007). Hepatoprotective and antioxidant effects of *Cuscuta chinensis* against acetaminophen-induced hepatotoxicity in rats. *Journal of Ethnopharmacology*.

[8] Liu J. H., Jiang B., Bao Y. M., An L. J. (2003). Effect of *Cuscuta chinensis* glycoside on the neuronal differentiation of rat pheochromocytoma PC12 cells. *International Journal of Developmental Neuroscience*.

[9] Jin S. Y., Wang Q. (2004). *Studies on Processing of Chinese Medicinal Yinpian and Its Clinical Application*.

[10] Wu H., Waldbauer K., Tang L. (2014). Influence of vinegar and wine processing on the alkaloid content and composition of the traditional Chinese Medicine Corydalis rhizoma (Yanhusuo). *Molecules*.

[11] Wu H., Hu C. J. (2012). *Chinese Medicine Processing Discipline*.

[12] Zhao Z. Z., Liang Z. T., Chan K. (2010). A unique issue in the standardization of Chinese materia medica: processing. *Planta Medica*.

[13] Chen J. M. (2009). *Enlightening Primer of Materia Medical*.

[14] Yan X. T. (1958). *Combinations of Materia Medical*.

[15] Wang H. J., Zhao J. J., Liu J. X. (2012). The pharmacological effects and its development prospect of semen cuscutae. *Acta Chinese Medicine and Pharmacology*.

[16] Ye M., Yan Y. N. (2000). The study on the pharmacological research of semen cuscutae. *Journal of Beijing University of TCM*.

[17] Chen M., Zhao L., Jia W. (2005). Metabonomic study on the biochemical profiles of a hydrocortisone-induced animal model. *Journal of Proteome Research*.

[18] Xu X. J., Xu L. Y., Guo Z. Y. (2014). Study on simultaneous determination of five kinds of flavonoids in seeds of semen cuscutae by HPLC. *World Chinese Medicine*.

[19] Yang S., Kuai D. P., Li S. S. (2015). The study on total flavonoids content comparison of *Cuscuta chinensis* and three differently processed products. *World Science and Technology/Modernization of Traditional Chinese Medicine and Materia Medica*.

[20] Cheng X.-L., Xu L.-Y., Wei F. (2014). Identification of raw cuscutae semen and its processed products by high performance liquid chromatography/diode-array detection/mass spectrometry (HPLC-DAD-MS) combined with principle component analysis. *Journal of Liquid Chromatography & Related Technologies*.

[21] Yang J., Wang Y., Bao Y., Guo J. (2008). The total flavones from *Semen cuscutae* reverse the reduction of testosterone level and the expression of androgen receptor gene in kidney-yang deficient mice. *Journal of Ethnopharmacology*.

[22] Yen F.-L., Wu T.-H., Lin L.-T., Cham T.-M., Lin C.-C. (2008). Concordance between antioxidant activities and flavonol contents in different extracts and fractions of Cuscuta chinensis. *Food Chemistry*.

[23] Lee M.-S., Chen C.-J., Wan L. (2011). Quercetin is increased in heat-processed Cuscuta campestris seeds, which enhances the seed's anti-inflammatory and anti-proliferative activities. *Process Biochemistry*.

[24] Son Y.-O., Lee K.-Y., Kook S.-H. (2004). Selective effects of quercetin on the cell growth and antioxidant defense system in normal versus transformed mouse hepatic cell lines. *European Journal of Pharmacology*.

